# Difficult Places, Unexpected Discoveries

**DOI:** 10.3201/eid2507.AC2507

**Published:** 2019-07

**Authors:** Byron Breedlove, J. Todd Weber

**Affiliations:** Centers for Disease Control and Prevention, Atlanta, Georgia, USA

**Keywords:** art science connection, emerging infectious diseases, art and medicine, about the cover, SciArt, Amie Esslinger, hydro vents and other difficult places, difficult places, unexpected discoveries, pathogens, microbes, bacteria, fungi, extreme conditions, antimicrobial drugs, antimicrobial resistance, promiscuous pattern realism

**Figure:**
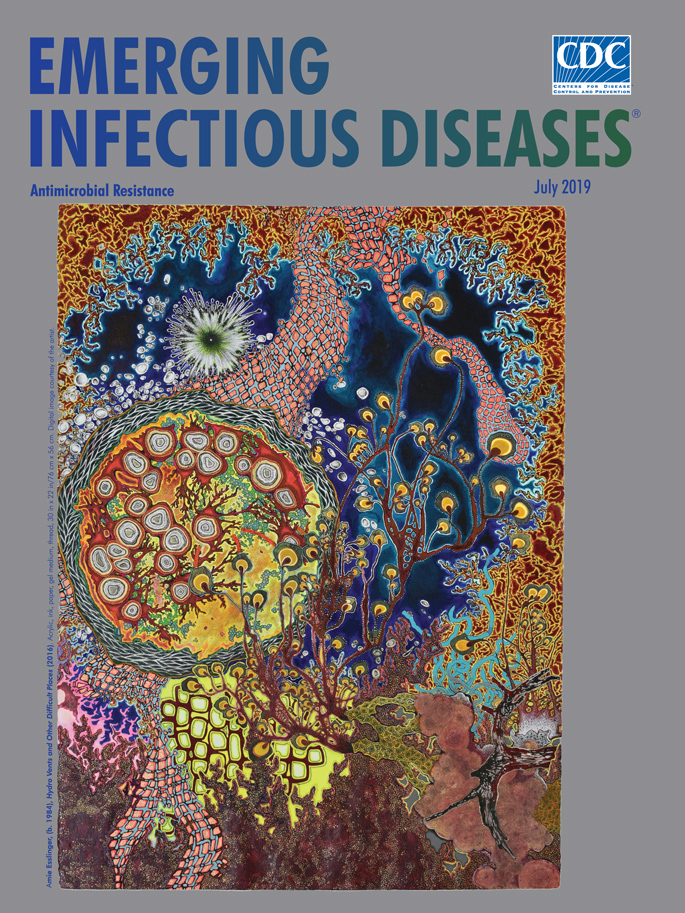
**Amie Esslinger (b. 1984), Hydro Vents and Other Difficult Places (2016)**. Acrylic, ink, paper, gel medium, thread, 30 in x 22 in/76 cm x 56 cm. Digital image courtesy of the artist.

Microbes, including myriad pathogens, have demonstrated their tenacity and malleability to endure, even flourish, under extreme conditions thought to be inhospitable to life. These microbes evolve at a pace that proves hard to fathom: they can undergo as many as 500,000 generations during a single human generation.

The proliferation and abundance of modern antibiotics have accelerated the pace of pathogens’ evolutionary adaptation through mutation and acquisition of genetic material conferring resistance from other species. The World Health Organization notes that new resistance mechanisms are emerging and spreading around the world and that without effective antimicrobials, treating infectious diseases is becoming increasingly challenging.

Researchers Julian and Dorothy Davies offer this perspective: “What happened during the evolution of bacteria and other microbes and organisms over several billions of years cannot be compared to the phenomenon of antibiotic resistance development and transfer over the last century. Contemporary selection pressure of antibiotic use and disposal is much more intense; selection is largely for survival in hostile environments rather than for traits providing fitness in slowly evolving populations.”

The concept of life thriving and adapting under extreme conditions resonates with Atlanta-based artist Amie Esslinger. Her painting “Hydro Vents and Other Difficult Places” appears as this month’s cover art. Esslinger explains that her approach “interjects activity not visible to the human eye into the real space of the gallery.” She works carefully and meticulously, creating complex, multilayered works based on “organic cell structures, aerial landscapes, and other hidden spaces.” Esslinger labels her work as “promiscuous pattern realism” and cites among her influences 19th century Victorian illustrations, in particular *Kunstformen der Natur* (*Artforms of Nature*) by Ernst Haeckel (A. Esslinger, pers. comm. email, 2019 May 16).

Esslinger explains what inspired her to create this painting. “Hydrothermal vents exist on the deepest sea floors. Under the cover of complete darkness, they emit magma, minerals, and chemicals while geothermally heating the water to temperatures often above the boiling point. At first blush, these vents would seem to create toxic, uninhabitable environments, yet, surprisingly, the areas around them are biologically more productive than elsewhere in the deep sea. Despite the extreme temperatures, high acidity, and lack of sunlight, microorganisms adapt and thrive there. This natural phenomenon surprises and excites me. I wanted to capture the hyperactivity involved in the survival and adaptation of the organisms in these seemingly contradictory environments” (A. Esslinger, pers. comm. email, 2019 May 16).

Her painting evokes the teeming life forms that surround deep ocean oases. Lines, shapes, and textures converge and overlap. Awash in colors and shapes, the overall visual effect suggests pulsation and displacement. A filigree of irregular yellow-rimmed, reddish-brown masses frames the top and right sides of the image, and a dense cacophony of speckled crimson rises from the bottom. Reddish ribbons of scaly cellular forms cluster together, and flowerlike forms float at the end of undulating tendrils. Wreathed by interlaced white and black lines, a collection of organisms resembling sliced geodes dominates the left center, fighting with the strange starburst just above it for the viewer’s attention. Cerulean-edged indigo seawater appears through this dense curtain of exotic shapes.

The artist notes that although she was not striving to portray actual life-forms realistically, elements in the painting are based on various bacteria and tubeworms. In her words, “I want to represent the vitality and potentiality of simple cell organisms. The piece is an expressive attempt to capture the activity and chance mutations that allow life to make these strange places home.” 

This issue of the journal lays out the diversity of those mutations and spread of antimicrobial resistance through environmental pressure and horizontal gene transfer with articles on *Aspergillus fumigatus*, *Campylobacter jejuni*, *Candida auris*, *Enterobacteriaceae*, ceftriaxone-resistant *Neisseria gonorrhoeae*, carbapenem-resistant *Pseudomonas aeruginosa*, tuberculosis, and the gut microbiome. This selected list overlaps with pathogens that can travel via water to cause infection (e.g., *Aspergillus fumigatus*, *Campylobacter*, *Escherichia coli*, and *Pseudomonas aeruginosa*), a list that further includes pathogens such as *Legionella pneumophila*, nontuberculous mycobacteria, and *Vibrio* spp., among others.

On the flip side, extreme conditions may also harbor solutions. Researchers have postulated that deep-sea organisms, perhaps similar to those depicted in “Hydro Vents and Other Difficult Places,” could be sources of novel bioactive compounds that could aid in the development of new antimicrobial drugs. Curiosity about the interactions between pathogens and cellular life forms in difficult places creates an interesting bridge between art and science and may lead to discoveries that could forge unexpected solutions to the global crisis of antimicrobial resistance.
